# Irisin alleviates obesity-related spermatogenesis dysfunction via the regulation of the AMPKα signalling pathway

**DOI:** 10.1186/s12958-021-00821-1

**Published:** 2021-09-08

**Authors:** Yang Mu, Huang-Guan Dai, Ling-Bo Luo, Jing Yang

**Affiliations:** 1 Reproductive Medicine Center, Renmin Hospital of Wuhan University, Wuhan, 430060 China; 2 Department of Reproductive Medicine, Yantai Yuhuangding Hospital, Affiliated Hospital to Qingdao University, Yantai, Shandong China

**Keywords:** Irisin, HFD, Spermatogenesis dysfunction, AMPKα

## Abstract

**Background:**

Infertility is a common complication in obese men. Oxidative stress and testicular apoptosis play critical roles in obesity-induced spermatogenesis dysfunction. It has been reported that irisin, an exercise-induced myokine, may attenuate oxidative damage and testicular apoptosis in several diseases; however, its role in obesity-induced spermatogenesis dysfunction remains unclear. The purpose of this study was to investigate the role and underlying mechanism of irisin in obesity-induced dysfunction of spermatogenesis.

**Methods:**

Male mice were fed a high-fat diet (HFD) for 24 weeks to establish a model of obesity-induced spermatogenesis dysfunction. To explore the effects of irisin, mice were subcutaneously infused with recombinant irisin for 8 weeks beginning at 16 weeks after starting a HFD. To confirm the role of AMP-activated protein kinase α (AMPKα), AMPKα-deficient mice were used.

**Results:**

The data showed decreased serum irisin levels in obese patients, which was negatively correlated with sperm count and progressive motility. Irisin was downregulated in the plasma and testes of obese mice. Supplementation with irisin protected against HFD-induced spermatogenesis dysfunction and increased testosterone levels in mice. HFD-induced oxidative stress, endoplasmic reticulum (ER) stress and testicular apoptosis were largely attenuated by irisin treatment. Mechanistically, we identified that irisin activated the AMPKα signalling pathway. With AMPKα depletion, we found that the protective effects of irisin on spermatogenesis dysfunction were abolished in vivo and in vitro.

**Conclusions:**

In conclusion, we found that irisin alleviated obesity-related spermatogenesis dysfunction via activation of the AMPKα signalling pathway. Based on these findings, we hypothesized that irisin is a potential therapeutic agent against obesity-related spermatogenesis dysfunction.

## Introduction

Infertility is a disease defined as the failure to establish a successful clinical pregnancy after at least 1 year of regular and unprotected sexual intercourse; presently, it affects approximately 8%-12% of reproductive-age couples [1]. Both female and male factors contribute to infertility, and male factors account for approximately half of all cases [2]. Obesity exerts adverse impacts on male fertility by affecting endocrine, thermal, genetic and sexual mechanisms and has been regarded as an important cause of male infertility [3, 4]. However, the mechanisms underlying obesity-related male infertility remain unclear.

Previous studies have demonstrated that reactive oxygen species (ROS)-mediated damage to spermatozoa is a major cause of male infertility and affects nearly 30%-80% of infertile men [5, 6]. Oxidative stress has adverse effects on both the functional and structural integrity of spermatozoa, which leads to sperm cell dysfunction and male infertility [7–10]. ROS induced by fat accumulation-related proteins attack lipids and membranes, form highly reactive 4-hydroxynonenal (4-HNE)-protein adducts in testicular tissues and affect cellular integrity [11, 12]. The increased incidence of generated oxidative stress caused by enhanced ROS production might be due to reduced ferric reducing antioxidant power (FRAP) activity and impaired antioxidant defence system [13, 14]. ROS produced as a result of obesity also have toxic effects on Leydig cell function [15–17]. In addition, increased levels of free radicals could cause endoplasmic reticulum (ER) stress, which is defined as misfolded proteins that accumulate in the lumen of the ER [18, 19]. Our previous study reported that ER stress played a key role in obesity-induced reproductive dysfunction [20, 21]. ER stress was activated in testes of obese mice, and attenuation of ER stress by pharmacological agents improved spermatogenic function in HFD-fed mice [20, 21].

Testicular apoptosis is an integral part of normal spermatogenesis and can be enhanced in certain disease conditions, such as obesity [22, 23]. Accumulating evidence has indicated that enhanced testicular apoptotic cell death, predominantly via ROS- and endoplasmic reticulum (ER) stress-mediated cell death pathways, plays a critical role in obesity-related male infertility [20, 21, 24]. ROS produced by excessive free fatty acids attack the cell membrane and damage DNA and cause DNA fragmentation, which activates Bax and caspase 3 signalling and induces testicular cell apoptosis [25, 26]. In addition, ER stress caused by obesity also promotes testicular apoptotic cell death via upregulation of the expression of C/EBP homologous protein (CHOP) [20, 21]. Therefore, finding a negative regulator of oxidative stress, ER stress and cell apoptosis in male testes is of vital importance for the clinical treatment of male infertility.

Irisin, a type I membrane protein, was first discovered as a peroxisome proliferator-activated receptor γ coactivator-1 (PGC-1) α-dependent myokine that can convert white adipose tissue to brown adipose tissue [27–29]. Irisin reduces gluconeogenesis via the phosphoinositide 3-kinase (PI3K)/protein kinase B (AKT)/glycogen synthase kinase 3 (GSK3) pathway [30]. Irisin also promotes browning of white adipocytes by activating p38 mitogen-activated protein kinase (MAPK) and extracellular-signal regulated kinase (ERK) pathways in adipocytes [31]. Recently, it has been reported that irisin could alleviate organ injuries by inhibiting oxidative stress and subsequent cell loss [32–34]. Although irisin has been shown to suppress apoptosis and reduce oxidative damage in cardiometabolic diseases [35, 36], irisin has not been further investigated for additional biological activity in the testis, especially in relation to obesity-related male infertility. Based on these findings, we hypothesized that irisin might be a promising candidate for the treatment of obesity-related spermatogenesis dysfunction. To the best of our knowledge, this is the first report of a protective role for irisin in obesity-related spermatogenesis dysfunction.

## Methods

### Reagents

Irisin (SRP8039), thapsigargin (TG, T9033), Akt1/2 kinase inhibitor (A6730), Ex527 (E7034), Compound C (CpC, P5499), and palmitic acid (PA) (P9767) were purchased from Sigma–Aldrich (St. Louis, MO, USA).

### Human study

Human semen samples as well as serum samples were collected from healthy lean males (control) and obese subfertile subjects who volunteered to participate in this project. All participants were male outpatients who went to the reproductive medicine centre of Renmin Hospital of Wuhan University (Wuhan, China) for medical care between April and October 2018. Informed consent was obtained before they took part in the project. A questionnaire was completed by each participant to collect information about ethnicity, age, height, body weight, medical history, and lifestyle, which was required for further group division. Men with other known causes of male subfertility, such as infection, varicocele, obstruction of the vas deferens, chromosomal abnormalities or smoking were excluded from this study. All participants were divided into two groups, the control group and obesity (BMI ≥ 30) group, according to their BMI. Sperm count and progressive motility were detected according to WHO guidelines. Serum samples were collected from abandoned blood samples. The human experiments carried out in this study conformed to the Declaration of Helsinki and were approved by the Human Research Ethics Committees of Renmin Hospital of Wuhan University in Wuhan, China.

### Animals and treatment

Male C57BL/6 J mice (age 8–10 weeks, body weight 20–26 g) were purchased from the Institute of Laboratory Animal Science, Chinese Academy of Medical Sciences (Beijing, China). The mice were housed in the experimental animal centre of Renmin Hospital of Wuhan University (less than 5 mice per cage, 20 °C–25 °C, 50% humidity, 12 h light/dark cycle). The animal experiments included in our study were performed according to the Guidelines for the Care and Use of Laboratory Animals published by the United States National Institutes of Health (NIH Publication, revised 2011) and the Guidelines for the Care and Use of Laboratory Animals of the Chinese Animal Welfare Committee and were approved by the Animal Use Committees of Renmin Hospital of Wuhan University. Mice were randomly allocated into the four groups (12 mice per group): (1) normal diet (ND) group, in which mice were fed a standard chow for 24 weeks and infused with normal saline for 8 weeks; (2) ND + irisin group, in which mice were fed a standard chow for 24 weeks and subcutaneously infused with recombinant irisin (12 nmol/kg/day) for 8 weeks beginning at 16 weeks of ND via osmotic minipumps as previously described [37, 38]; (3) high-fat diet (HFD) group, in which mice were fed a HFD for 24 weeks and infused with normal saline for 8 weeks; (4) HFD + irisin group, in which mice were fed a HFD as well as given irisin (12 nmol/kg/day) infusions for 8 weeks. The HFD composition was reported in a previous study [39]. In addition, irisin dosage was determined according to a previous study [35]. After that, the mice were sacrificed, and testes, epididymis and blood samples were collected for further analysis. Body weight and testes weight were recorded for further analysis.

To further verify the role of AMPKα, AMPKα global knockout mice were also fed a HFD and given irisin infusions as described above. The source of AMPKα global knockout mice has been described previously [40]. The littermates were used as the controls.

### Cell culture and treatment

Mouse sperm were cultured in G-IVF (Vitrolife, Sweden) medium at a concentration of 5 × 10^6^ spermatozoa/ml. TM3 mouse Leydig cells were cultured in DMEM/F12 medium containing 10% foetal bovine serum (FBS). The two types of cells were treated with irisin (20 nmol/L) in the presence or absence of fatty medium (1 mmol/L, PA) for 24 h. To mimic the redox status or ER stress in testes of obese mice, the two cell types were treated with hydrogen peroxide (HP, 200 μmol) or thapsigargin (Tg, 1 mol/l) for 6 h [41, 42]. To explore the mechanism through which irisin provided protection, the two cell types were incubated with CpC (20 μmol/L, an AMPK inhibitor), an Akt1/2 kinase inhibitor (1 μmol/L) or Ex527 (a specific Sirt1 inhibitor, 1 μmol/L) for 12 h.

### Serum irisin, glucose, insulin and cholesterol level detection

Blood was collected from the retro-orbital plexus of anaesthetized mice at 24 weeks after being fed a HFD. Serum was separated from mouse blood by centrifugation for 15 min at 1000 g. Fresh serum was used to detect serum irisin, glucose, insulin and cholesterol levels. Irisin Competitive ELISA Kit was provided by AdipoGen LIFE SCIENCES (Boppard, Germany). The detection procedure was performed according to the manufacturer’s instructions as previously described [43]. The serum insulin level was determined by an Insulin ELISA kit (Demeditec Diagnostics DmbH, Hamburg, Germany) according to the manufacturer’s instructions. An automatic biochemistry analyser (CX4/Pro, Beckman, CA, USA) was used to determine serum cholesterol levels.

### Serum hormone detection

Fresh serum was used to detect hormone levels. Mouse luteinizing hormone (LH, ab235648) levels were measured using the Mouse LH Beta ELISA Kit (ab235648) provided by Abcam (Cambridge, UK). This kit is a sandwich ELISA designed for the quantitative measurement of LH protein in tissue extracts. The intensity was measured at 450 nm with a fluorescence microplate reader. A mouse follicle stimulating hormone (FSH) ELISA Kit (abx154039) and mouse free testosterone ELISA Kit (abx254089) were purchased from Abbexa (Cambridge, UK). Mouse LH, FSH and testosterone were detected using these kits according to the manufacturer’s instructions as previously described [44, 45]. Serum samples and biotin-conjugated reagent were added to antibody-precoated wells. After that, the HRP-conjugated reagent and TMB substrate were then added. The intensity of the yellow colour was measured spectrophotometrically at 450 nm with a fluorescence microplate reader.

### Semen analysis in mice

The epididymis was carefully separated from testicular fat and subsequently put into Ringer’s solution and dissected. Then, the sperm count, viability and motility were measured according to a previously described protocol [20]. The sperm number was counted with a haemocytometer independently three times by two authors without knowledge of group assignment.

An eosin-nigrosin dye exclusion test was performed to assess sperm viability. Briefly, sperm were incubated with eosin-nigrosin staining solution and smeared on a microscope slide. After that, sperm were observed under a light microscope. The viable cells remained unstained, while the nonviable cells took up the stain [46].

Aliquots of sperm were incubated in Ringer’s solution and placed into computer-assisted sperm analysis (CASA, Microptic, Barcelona, Spain) assay chambers, and motility was examined on a 37 °C microscopic stage under a 10 × phase contrast objective (Nikon, Japan). Images were recorded and analysed [47].

### Histological analysis

Testis tissue samples were fixed in Bouin’s solution, dehydrated, and embedded in paraffin according to our previous study [21]. Haematoxylin and eosin (HE) staining of testis tissues was performed to assess morphology. Histological changes were evaluated by two researchers unaware of group assignment using a light microscope.

### Western blot analysis

Testis tissues were lysed in RIPA buffer, and total proteins were extracted according to previous studies [37, 38]. Protein concentration was detected with a BCA Protein Assay Kit according to the manufacturer’s instructions. Proteins were separated with SDS–PAGE gels and transferred to PVDF membranes. After blocking with 5% skimmed milk, membranes were incubated overnight at 4 °C with the following primary antibodies: rabbit anti-FNDC5 antibody (ab174833, Abcam, Cambridge, UK, 1:1000 dilution); rabbit anti-CHOP antibody (ab10444, Abcam, 1:500 dilution), rabbit anti-glucose-regulated protein 78 (GRP78) antibody (ab21685, Abcam, 1:500 dilution), rabbit anti-phosphor-protein kinase R-like ER kinase (PERK) antibody (ab192591, 1:500, dilution), rabbit anti-PERK antibody (ab79483, Abcam, 1:1000 dilution), rabbit anti-phosphor-eukaryotic translation initiation factor 2α (eIF2α) antibody (ab227593, Abcam, 1:1000 dilution), rabbit anti-eIF2α antibody (ab169528, Abcam, 1:1000 dilution), rabbit anti-Bad antibody (ab32445, Abcam, 1:1000 dilution), rabbit anti-Bax antibody (ab182733, Abcam, 1:500 dilution), rabbit anti-phosphor-AMPKα antibody (ab133448, Abcam, 1:1000 dilution), rabbit anti-AMPKα antibody (ab131512, Abcam, 1:1000 dilution), rabbit anti-phosphor-Acetyl-CoA carboxylase (ACC) antibody (Cell Signaling Technology, 11,818, 1:500 dilution), rabbit anti-ACC antibody (Cell Signaling Technology, 3676, 1:500), rabbit anti-nuclear factor E2-related factor 2(Nrf2) antibody (ab92946, Abcam, 1:1000 dilution), rabbit anti-heme oxygenase-1 (HO-1) antibody (ab189491, Abcam, 1:1000 dilution), rabbit anti-NADPH quinineoxidoreductase-1 (NQO-1) antibody (ab80588, Abcam, 1:1000 dilution), and rabbit anti-GAPDH antibody (ab8245, Abcam, 1:5000 dilution). After being washed three times, membranes were incubated with secondary antibodies for 1 h at room temperature. Finally, the membranes were examined using a chemiluminescence ECL kit (Bio–Rad, USA) and scanned by Image Lab 5.2.1. The protein expression was normalized to GAPDH expression.

### RNA extraction and gene expression analysis

Total RNA was extracted from testis tissues using TRIzol reagent. The mRNA was subsequently reverse transcribed to cDNA using a Transcriptor First Strand cDNA Synthesis Kit (Roche, Basel, Switzerland). Gene expression was analysed with real-time PCR using LightCycler 480 SYBR Green 1 Master Mix (Roche). The primers were synthesized by Takara Biomedical Technology (Beijing, China).

### Detection of caspase 3 and SOD activity

Caspase-3 activity and SOD activity were measured using commercialized detection kits according to the manufacturer’s instructions. The Caspase-3 Activity Assay Kit (Fluorometric) was provided by Abcam. A total superoxide dismutase (T-SOD) assay kit (hydroxylamine method) was provided by Nanjing Jiancheng Bioengineering Institute (Nanjing, China). The detection protocols were performed according to a previous article [35].

### Measurement of ROS generation and 4-hydroxynonenal

Spermatozoa were separated from seminal plasma by centrifugation for the detection of ROS by 2,7-dichlorofluorescin diacetate (DCFH-DA) as described previously [48]. Cells were incubated with DCFH-DA (10 μmol/L) at 37 °C for 60 min, and ROS production was determined by a fluorescence microplate reader. 4-Hydroxynonenal (4-HNE)-protein adducts were detected using a Lipid Peroxidation (4-HNE) Assay Kit (Abcam, ab238538). This competitive ELISA kit allowed detection of the 4-HNE adduct in testis samples determined by comparing its absorbance with that of a known 4-HNE-BSA standard curve.

### TUNEL analysis

Briefly, paraformaldehyde-fixed, paraffin-embedded testis sections were analysed by transferase-mediated deoxyuridine triphosphate-biotin nick end labelling (TUNEL) according to the manufacturer’s instructions. The sections were observed under a fluorescence microscope (BX51, Olympus, Japan). A total of 25 fields were randomly selected in each group (5 fields/mouse), and 100 cells were counted in each field.

### Statistical analysis

Data were analysed with SPSS 22.0 software. Single comparisons between two groups were performed using a t-test. Multiple comparisons among groups were analysed by one-way ANOVA followed by post hoc LSD tests. The correlation between two factors was assessed by Spearman’s analysis. All the measurement data in this study are expressed as the means ± SEM, and statistical significance is denoted as *p* < 0.05.

## Results

### Irisin was downregulated in obese patients and mice

**Fig. 1:**
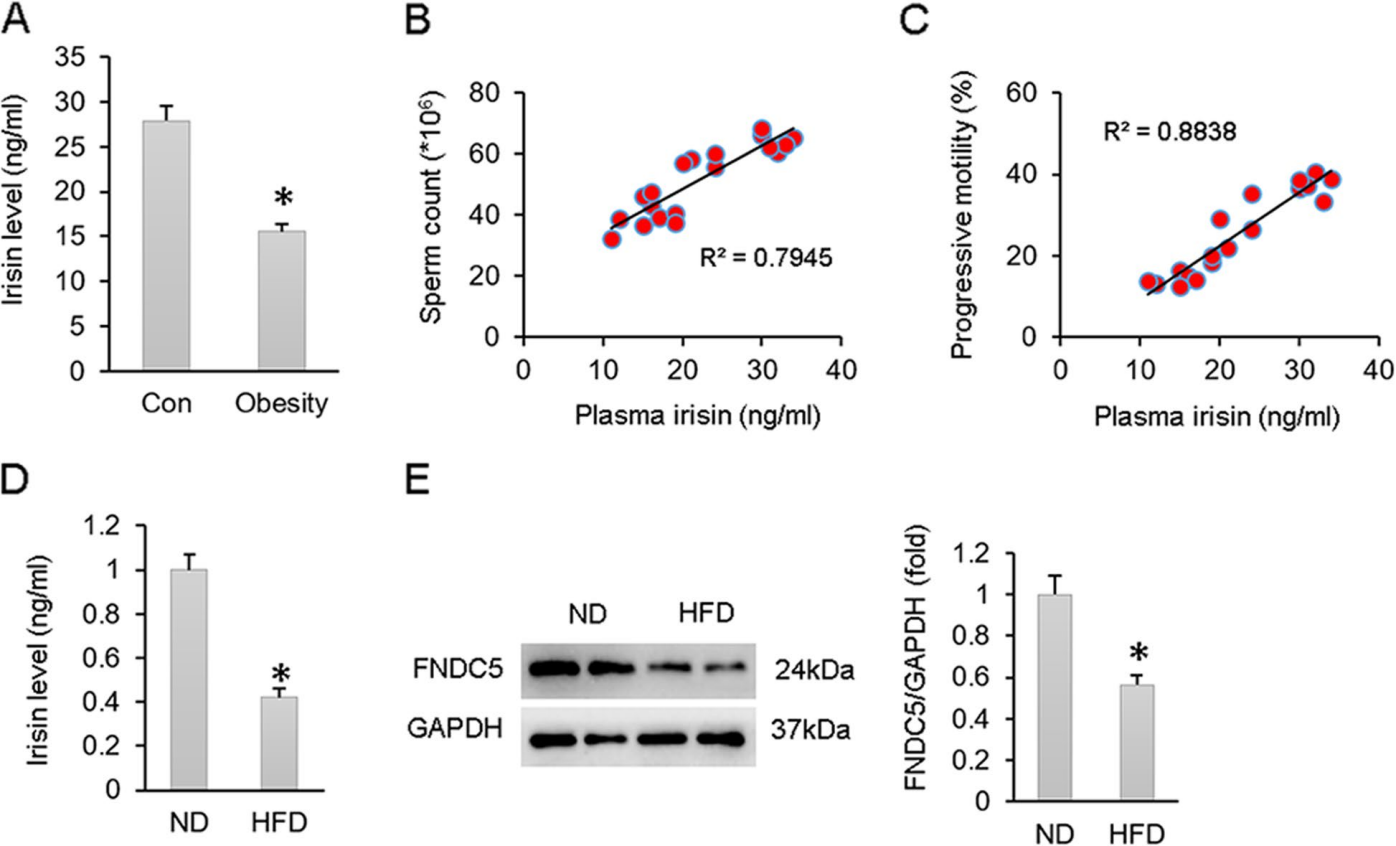
Irisin expression is downregulated by obesity. **A** The serum irisin levels of obese and control humans (Con: *n* = 10; Obesity: *n* = 9). **B** Correlation between sperm count and plasma irisin level (Con: *n* = 10; Obesity: *n* = 9). **C** Correlation between progressive sperm rate and plasma irisin level (Con: *n* = 10; Obesity: *n* = 9). **D** Serum irisin level of HFD mice and ND mice (*n* = 6). **E** FNDC5 expression in the testes of HFD mice and ND mice (*n* = 6). Data are presented as the mean ± SEM. ^*^P < 0.05 vs. the matched control

To elucidate the role of irisin in obesity-induced male infertility, we first measured irisin levels in obese patients. The characteristics of the patients and the sperm parameters are listed in Table 1. As shown, sperm count, progressive motility and serum testosterone levels were decreased in obese subfertile patients compared with those in normal lean males (Table 1). Plasma irisin levels were decreased in obese male subjects (Fig. 1A). Correlation analysis revealed that plasma irisin levels were negatively correlated with sperm count and progressive motility (Fig. 1B-C). The results from animal experiments also demonstrated that serum irisin levels and testicular FNDC5 protein expression were decreased in HFD-fed male mice (Fig. 1D-E).

**Table 1:** Characteristics of the indicated groups

Group	Sample size (n)	Age (years)	BMI (kg/m^2^)	Sperm count (*10^6^)	Progressive motility (%)	Testosterone (ng/mL)	FSH (mIU/mL)	LH (mIU/mL)
Control	10	27.9 ± 0.9	20.92 ± 0.45	61.04 ± 1.15	33.29 ± 1.97	4.18 ± 0.15	4.72 ± 0.24	3.68 ± 0.19
Obesity	9	28.9 ± 2.1	32.42 ± 0.46*	41.18 ± 1.28*	15.53 ± 0.83*	2.79 ± 0.12*	4.95 ± 0.23	3.37 ± 0.14

Date are expressed as the mean ± SEM

^*^p < 0.05, vs. Control group

### Supplementation with irisin attenuated HFD-induced spermatogenesis dysfunction

**Fig. 2:**
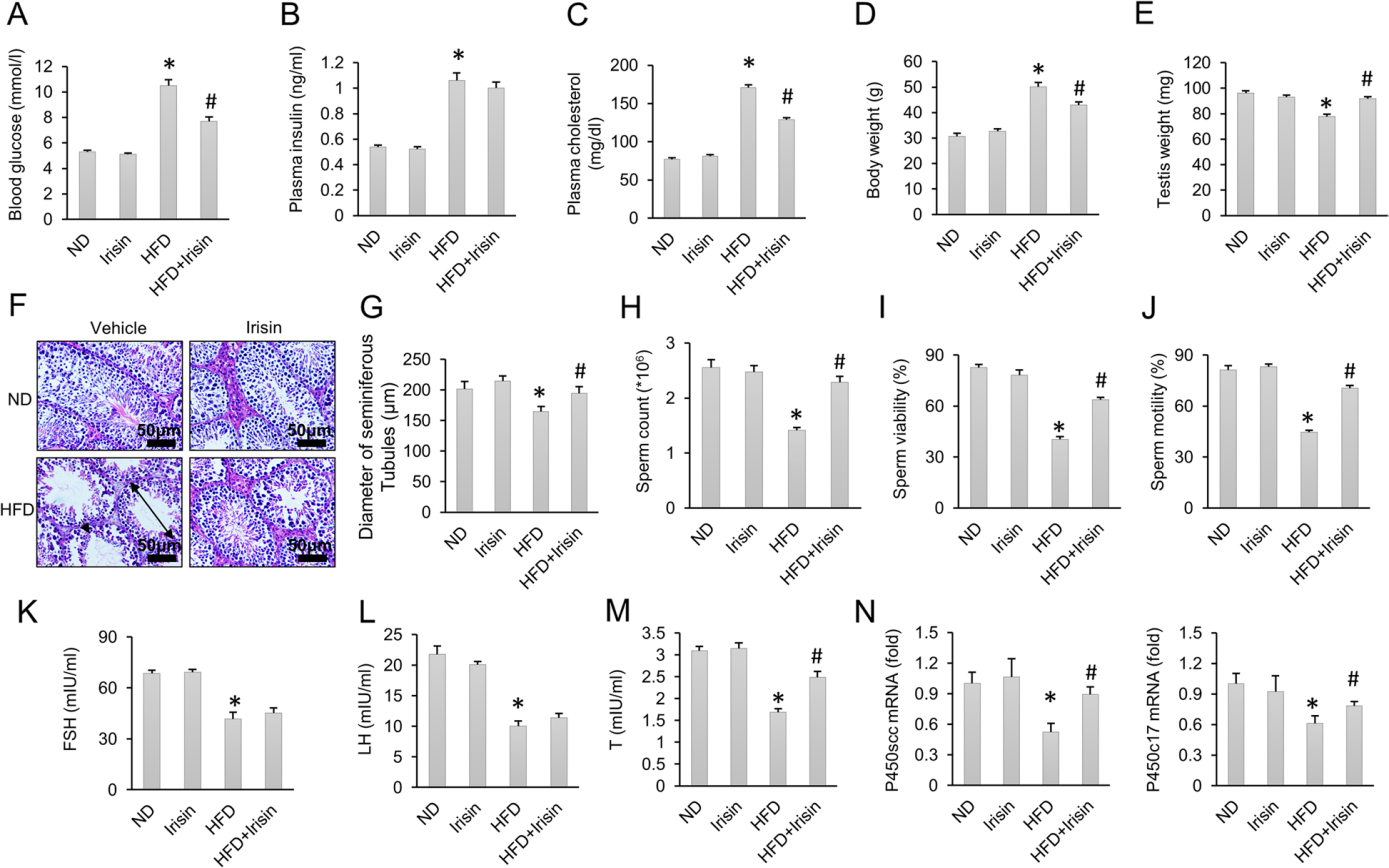
HFD-induced male spermatogenesis dysfunction was improved by irisin treatment. **A** Blood glucose level (*n* = 12). **B** Plasma insulin level (*n* = 12). **C** Plasma cholesterol level (*n* = 12). **D**-**E** Body weight and testes weight of the indicated groups (*n* = 12). **F** HE staining of mice testes. **G** Statistical analysis of the diameter of seminiferous tubules in the four groups (*n* = 6). **H**-**J** Sperm count, sperm viability and sperm motility (*n* = 6). **K**-**M** Serum FSH, LH and testosterone levels of the indicated groups (*n* = 6). **N** The mRNA expression of P450scc and P450c17 (*n* = 6). Data are presented as the mean ± SEM. ^*^P < 0.05 vs. the matched control. Atrophied seminiferous tubules are marked with a double-head arrow. Impairment of the blood-testis barrier in the testis is marked with an arrowhead

To explore the role of irisin in HFD-induced spermatogenesis dysfunction, irisin was infused into mice by osmotic micropump, and spermatogenesis function was evaluated. As shown in Fig. 2A-B, plasma glucose and insulin levels were increased in obese mice. After irisin treatment, plasma glucose and insulin levels were both decreased compared with those of the HFD-only group. Plasma cholesterol analysis also revealed that the increased plasma cholesterol level was reduced by irisin treatment (Fig. 2C). The increase in body weight of HFD-fed mice was decreased by irisin treatment (Fig. 2D). Next, the effect of irisin on HFD-induced spermatogenesis dysfunction was evaluated. The results demonstrated that testes weight was decreased in HFD-fed mice and this decrease was lessened by irisin administration (Fig. 2E). In the ND groups, testicular germ cells appeared normal, and the blood-testis barrier was intact. HFD treated mice exhibited atrophied seminiferous tubules with fewer sperm observed in testes of obese mice. The blood-testis barrier in these obese mice appeared to be thin and disorganised (Fig. 2F). Irisin treatment attenuated HFD-induced pathological alterations, as evidenced by the increased diameter of the seminiferous tubules and improved blood-testis barrier (Fig. 2F-G). Consistent with histologic staining, sperm quality analysis suggested that sperm count, sperm viability and sperm motility were all decreased in obese mice. After irisin supplementation, the decreased sperm count, sperm viability and sperm motility were all improved (Fig. 2H-J). Irisin treatment did not alter the decreased levels of FSH and LH in obese mice (Fig. 2K-L). Serum testosterone levels were significantly decreased by HFD but largely improved by irisin treatment (Fig. 2M). Next, we detected the mRNA levels of steroidogenic enzymes, including cholesterol side-chain cleavage P450 (P450scc) and 17α-hydroxylase/C17-20 lyase (P450c17). The data in our study showed that the pathological alterations in P450scc and P450c17 mRNA levels in obese mice were inhibited by irisin treatment (Fig. 2N).

### Irisin protected testes from oxidative damage in obese mice

**Fig. 3:**
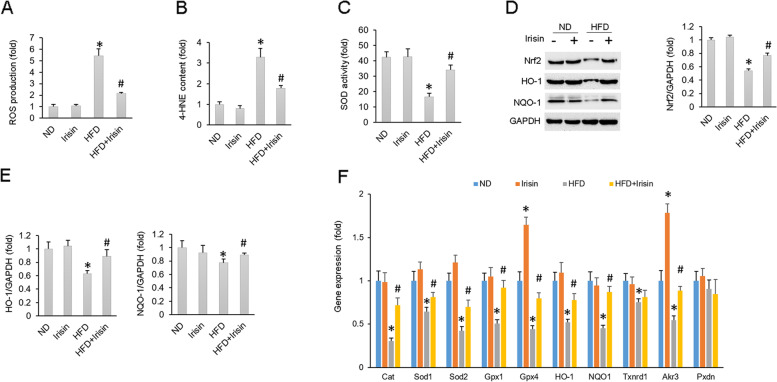
Oxidative stress was suppressed by irisin. **A** Testicular ROS production (*n* = 6). **B** Testicular A-HNE content (*n* = 6). **C** SOD activity in mice testes (*n* = 6). **D**-**E** Protein levels of Nrf2, HO-1 and NQO-1 (*n* = 6). **F** Oxidative stress-related gene expression in mouse testes (*n* = 6). Data are presented as the mean ± SEM. ^*^P < 0.05 vs. the matched control

HFD resulted in increased ROS production in spermatozoa; however, irisin treatment largely inhibited ROS production (Fig. 3A). 4-HNE is a byproduct of lipid peroxidation and is widely recognized as a stable marker of oxidative stress. A HFD resulted in increased 4-HNE in the testes, while irisin treatment significantly inhibited this pathological alteration (Fig. 3B). Irisin treatment also increased the total SOD activity in testes of obese mice (Fig. 3C). Nrf2 is a transcription factor that participates in the regulation of cellular redox balance and detoxification response in mammals [49, 50]. Western blot analysis showed that irisin treatment significantly increased Nrf2 expression in testes of obese mice (Fig. 3D-E). Detection of Nrf2-regulated downstream enzymes found that the decreased HO-1 and NQO1 protein expression in testes of obese mice was upregulated after irisin treatment (Fig. 3E–F). Further detection of transcript levels specifically for catalase (Cat), Sod1, Sod2, glutathione peroxidase 1 (Gpx1), Gpx4, HO1, NQO1, thioredoxin reductase 1 (Txnrd1), aldo–keto reductase 3 (Akr3) and peroxidasin (Pxdn) revealed that irisin treatment restored the levels of all the transcripts except Pxdn in testes of obese mice (Fig. 3F).

### Treatment with irisin decreased ER stress and cell apoptosis induced by a HFD

**Fig. 4:**
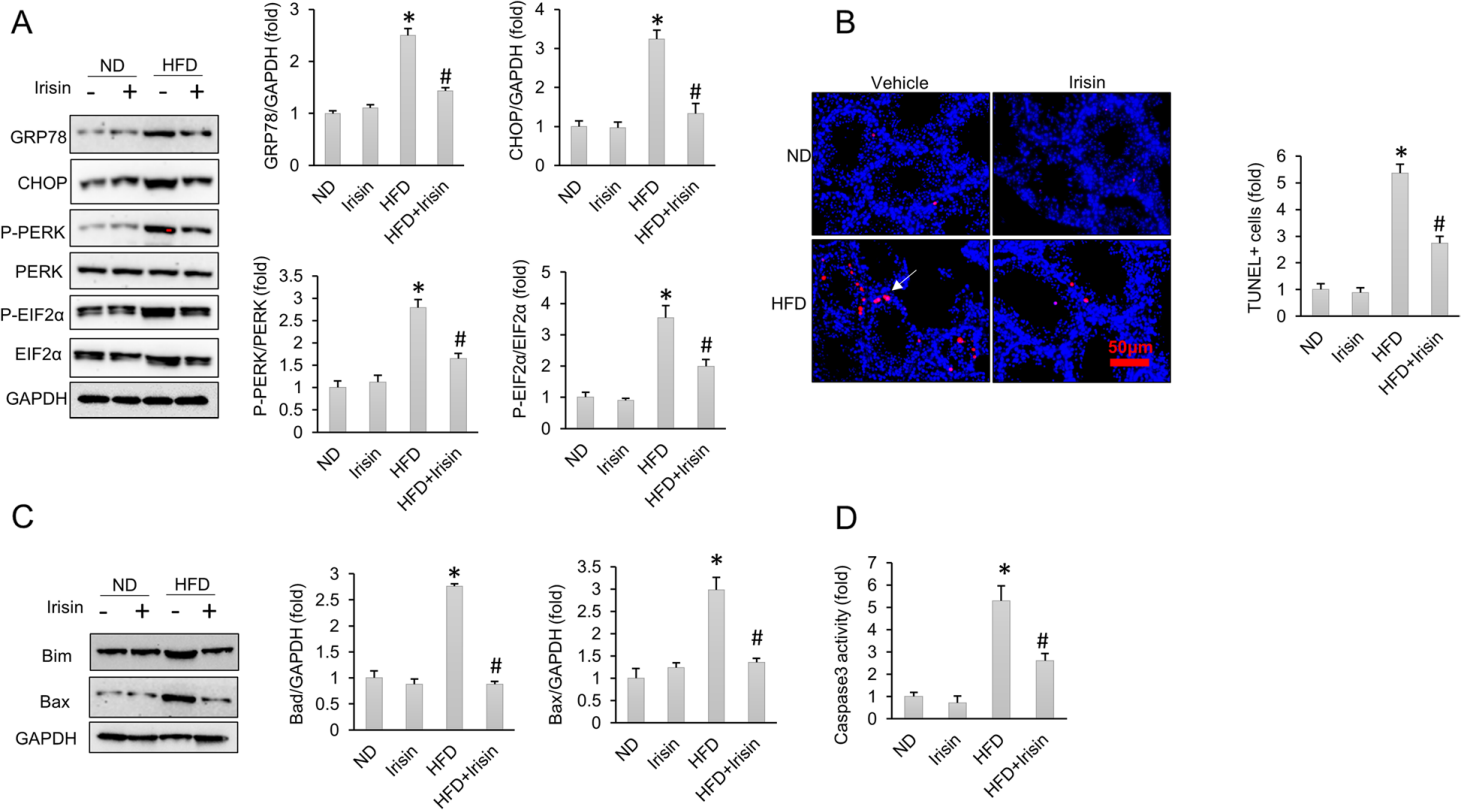
ER stress and cell apoptosis were attenuated by irisin. **A** Protein level of ER stress related markers (*n* = 6). **B** TUNEL staining of mice testes (*n* = 5). **C** Protein levels of Bim and Bax in mouse testes (*n* = 6). **D** Testicular caspase 3 activity (*n* = 6). Data are presented as the mean ± SEM. ^*^P < 0.05 vs. the matched control

As demonstrated in Fig. 4A, protein expression of GRP78, CHOP, p-PERK and p-EIF2α was upregulated by a HFD, and these pathological elevations were suppressed by irisin treatment. ER stress activation can trigger a proapoptotic response [51]. TUNEL staining revealed that testicular apoptosis was increased by a HFD and reduced by irisin treatment (Fig. 4B). The inhibitory effects of irisin on cell apoptosis were further confirmed by western blot results showing that irisin decreased the expression of Bim and Bax (Fig. 4C). Testicular caspase-3 activity analysis demonstrated that HFD-induced elevation in caspase-3 activity was attenuated by irisin (Fig. 4D).

### The protective effects of irisin on sperm function and testosterone production are mediated by the AMPKα signalling pathway

**Fig. 5:**
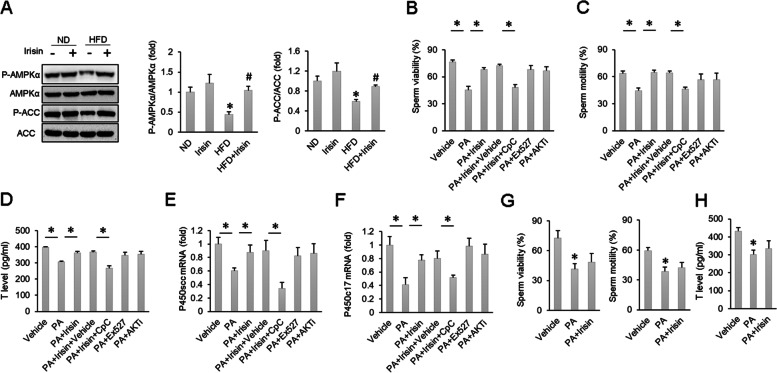
The protective effects of irisin on HFD-induced spermatogenesis dysfunction were abolished after AMPK inhibition. **A** Protein level of AMPK and ACC (*n* = 6). **B**-**C** Sperm viability and sperm motility of the indicated groups (*n* = 6). **D**-**F** T level and mRNA expression of P450scc and P450c17 (*n* = 6). **G**-**H** Sperm viability, sperm motility and T levels after AMPK knockout (*n* = 6). Data are presented as the mean ± SEM. ^*^P < 0.05 vs. the matched control

AMPKα has been reported to be a negative regulator of ER stress and cell apoptosis [31]. We detected phosphorylated AMPKα and ACC, a substrate of AMPKα. The results indicated that phosphorylated AMPKα and ACC were preserved in mice given irisin compared with those in the HFD group without irisin (Fig. 5A). A previous study reported that irisin could exert its protective effects via AKT activation [35]. Li et al. also found that irisin improved sepsis-related alveolar epithelial barrier dysfunction by activating sirtuin1 (Sirt1) pathways [52]. To further explore which signalling pathway was responsible for the protection provided by irisin, sperm were isolated and subjected to an AMPKα inhibitor (CpC), a Sirt1 inhibitor (Ex527) and an AKT inhibitor. Our data demonstrated that decreased sperm viability and motility in response to PA treatment were improved after irisin treatment and that the actions of irisin were abolished by CpC but not Ex527 or the AKT inhibitor (Fig. 5B-C). To confirm the role of AMPKα in the actions of irisin on testosterone production, we used TM3 mouse Leydig cells. Testosterone levels secreted by TM3 cells were decreased in response to PA but increased by irisin treatment (Fig. 5D). This effect of irisin was largely blocked by the use of CpC but not Ex527 or the AKT inhibitor (Fig. 5D). Further detection of the mRNA levels of steroidogenic enzymes revealed that the increased mRNA levels of P450scc and P450c17 due to irisin treatment were all reduced by CpC treatment (Fig. 5E–F). However, Ex527 or the AKT inhibitor did not offset the protective effect of irisin on the levels of P450scc and P450c17 (Fig. 5E–F). To further confirm the role of AMPKα in the protection provided by irisin, we isolated sperm and Leydig cells from AMPKα-deficient mice. The results indicated that irisin lost its protective effects on sperm viability, sperm motility and testosterone levels in PA-treated cells (Fig. 5G-H).

### Irisin suppressed oxidative damage and ER stress in an AMPKα-dependent manner in vitro

**Fig. 6:**
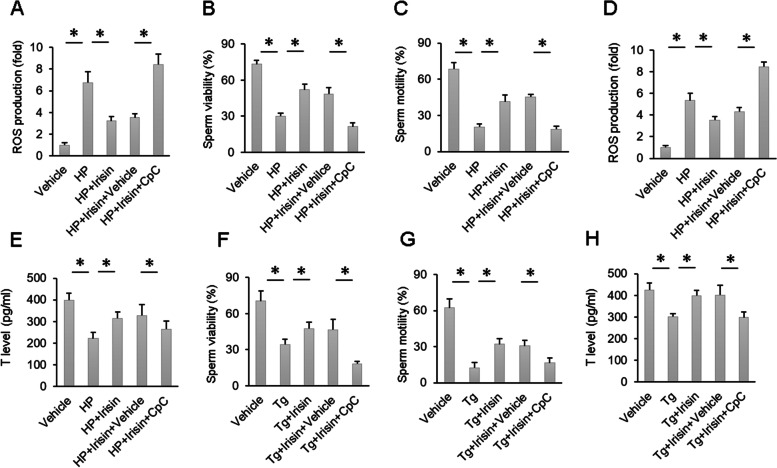
HP- and Tg-induced sperm damage and ROS production and a reduction in T levels were ameliorated by AMPK inhibition. **A** ROS production of sperm in the indicated groups (*n* = 6). **B**-**C** Sperm viability and sperm motility of the indicated groups (*n* = 6). **D** ROS production of Leydig cells in the indicated groups (*n* = 6). **E** T production of Leydig cells in the indicated groups (*n* = 6). **F**-**G** Sperm viability and sperm motility of the indicated groups (*n* = 6). **H** T production of Leydig cells in the indicated groups (*n* = 6). Data are presented as the mean ± SEM. ^*^P < 0.05 vs. the matched control

To further clarify that the protective effects of irisin on oxidative damage and ER stress were mediated by AMPKα, we incubated sperm with hydrogen peroxide (HP), which is a potent inducer of oxidative damage. As expected, HP-induced ROS production was largely prevented by irisin, and this effect was blocked by CpC (Fig. 6A). Irisin treatment also reduced oxidative damage induced by HP, as reflected by the alterations in sperm viability and motility, and lost its inhibitory effects after CpC treatment (Fig. 6B-C). Similar to findings in sperm, irisin suppressed the production of ROS and increased the secretion of testosterone in Leydig cells, and these actions of irisin were abolished by CpC (Fig. 6D-E). To further substantiate the effects of irisin on ER stress, we incubated sperm and Leydig cells with Tg, which is a potent inducer of ER stress. Tg induced impairment of sperm function as well as a reduction in testosterone production (Fig. 6F–H). Irisin exerted protective effects on the above pathological changes, and the protective effects of irisin were completely blocked by CpC (Fig. 6F–H).

### AMPKα depletion counteracted the protective effects of irisin in vivo

**Fig. 7:**
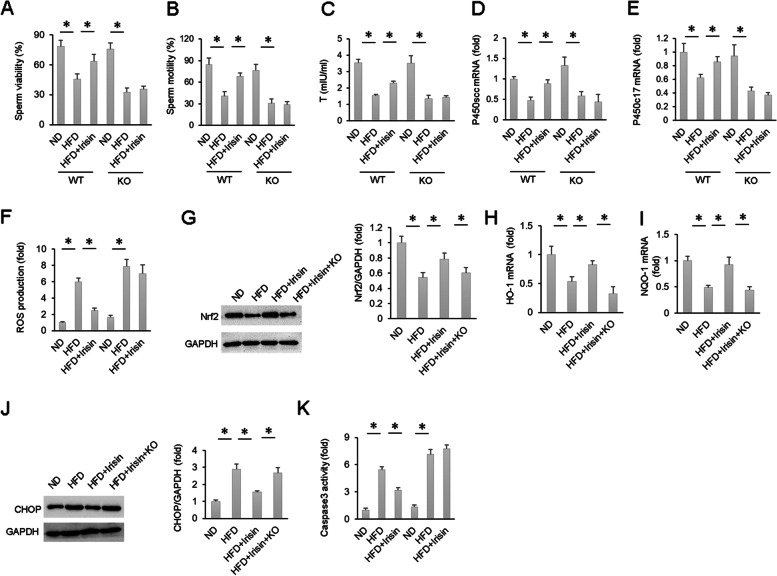
The protective effects of irisin on spermatogenesis function were abolished after AMPK knockout. **A**-**B** Sperm viability and sperm motility of the indicated groups (*n* = 6). **C**-**E** T level and mRNA expression of P450scc and P450c17 (*n* = 6). **F** ROS production of the indicated groups (*n* = 6). **G** Nrf2 expression in mice testes (*n* = 6). **H**-**I** mRNA levels of HO-1 and NQO-1 in mouse testes (*n* = 6). **J** CHOP expression in mice testes (*n* = 6). **K** Testicular Caspase-3 activity of the indicated groups (*n* = 6). Data are presented as the mean ± SEM. *P < 0.05 vs. the matched control

Subsequently, we further confirmed the key role of AMPKα in irisin-related testicular protection using AMPKα-deficient mice. The results suggested that the improvements in sperm viability and motility by irisin were prevented by AMPK deletion in obese mice (Fig. 7A-B). Irisin increased testosterone production and mRNA expression of P450scc and P450c17 in obese mice (Fig. 7C-E). These effects of irisin were abolished after AMPKα deficiency (Fig. 7C-E). The inhibitory effects of irisin on ROS production were also abolished by AMPKα deficiency (Fig. 7F). Subsequent detection revealed that the restoration of Nrf2 and downstream genes produced by irisin treatment was eliminated in AMPKα deficient mice (Fig. 7G-I). The protective effect of irisin against CHOP and Caspase 3 activity were also offset after AMPKα depletion (Fig. 7J-K).

## Discussion

**Fig. 8:**
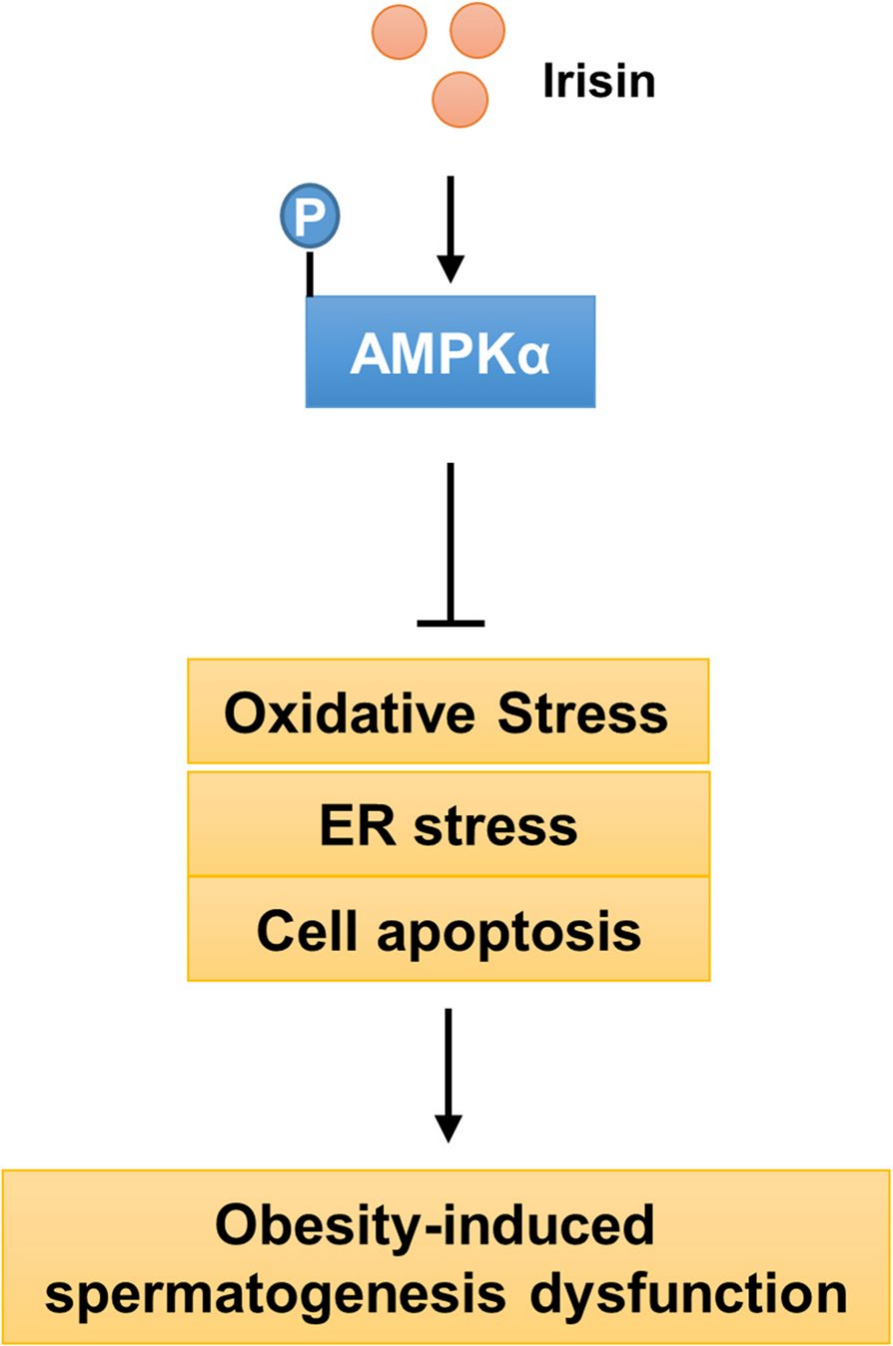
Schematic diagram of the molecular mechanisms underlying irisin-mediated protection of spermatogenic functions in mice

Previous studies have demonstrated the downregulation of irisin in obese rats compared with control groups, and that the overexpression of irisin could improve glucose/lipid metabolism in obese individuals [53, 54]. In our study, the circulating levels of irisin in obese humans and obese mice were decreased, which was consistent with a previous report [35]. Recent studies also found correlations between irisin levels and insulin resistance, nonalcoholic fatty liver disease and subclinical atherosclerosis [55]. In our study, we found that plasma irisin levels were negatively correlated with sperm count and sperm motility. Further detection revealed that FNDC5, which could be cleaved and released as irisin, was significantly decreased in obese mice. The above findings implicated irisin in the regulation of obesity-related impairment of spermatogenesis. Recently, Wahab et al. found that irisin was a novel endocrine factor involved in the regulation of spermatogonial activities [56]. However, there are no available data regarding the role of irisin in obesity-related spermatogenesis dysfunction. Here, we found that irisin supplementation alleviated oxidative stress and ER stress, thus inhibiting testicular cell apoptosis and improving spermatogenesis in obese mice. The findings in our study suggested that irisin might be a promising target for improving male reproductive ability and might be helpful for devising future therapies for male infertility (Fig. 8).

Oxidative stress has been regarded as an important pathological factor in male infertility. Low levels of ROS are necessary for sperm capacitation, hyperactivation, acrosomal reaction, and sperm-ovule fertilization [57]. However, high levels of ROS are harmful to male reproductive ability. ROS can attack the cell membrane, causing poor sperm quality and male infertility [10, 58, 59]. Higher levels of ROS in obese mice have been shown to disturb the reproductive function of males through lipid peroxidation, testicular cell apoptosis, and sperm DNA fragmentation [60, 61]. ROS attack sperm DNA and facilitate testicular apoptosis in a caspase-3-dependent manner [62]. The testis is weak in antioxidant defences, rendering it highly vulnerable to oxidative damage. Malondialdehyde and 4-HNE, the products of the reaction between ROS and biomembranes, could deplete the expression and decrease the activity of these low-expressed antioxidant enzymes, which further increases ROS-mediated genotoxicity and cellular toxicity [61, 62]. Previous studies have revealed that irisin could decrease oxidative stress in liver ischaemia–reperfusion injury [32]. FNDC5 overexpression alleviated oxidative stress and cardiomyocyte apoptosis in doxorubicin-induced cardiotoxicity [35]. In accordance with these previous studies, we found that oxidative stress induced by HFD was alleviated by irisin. Irisin decreased ROS production and reduced the byproduct of lipid peroxidation (4-HNE) but restored the activity of SOD in obese mice. Nrf2 is a transcription factor that can regulate the transcription of genes encoding protective molecules [63]. In response to ROS, Nrf2 is activated and regulates the expression of several cytoprotective proteins. Nrf2 deficiency causes age-dependent testicular oxidative stress, which disrupts spermatogenesis, indicating a critical role of Nrf2 in preventing oxidative disruption of spermatogenesis [64]. Consistent with these data, we found that irisin supplementation significantly increased Nrf2 expression and downstream targets. These data suggest that irisin may stimulate the production of Nrf2-mediated downstream antioxidants to protect against obesity-induced testicular damage.

Oxidative stress is also known to induce ER stress. It has been reported that ER stress is closely involved in PM 2.5-induced reproductive toxicology in male rats [65]. Our previous studies found that attenuation of ER stress by a pharmacological agent significantly reduced obesity-related testicular damage and improved spermatogenesis [20]. In line with these findings, we found that ER stress markers were increased in the testes of HFD-fed mice but decreased in irisin-infused mice. The suppression of ER stress might partly explain the protective effects of irisin against obesity-related spermatogenesis dysfunction. Oxidative stress and ER stress contribute to testicular apoptotic cell death [20]. As expected, a HFD was associated with a significant increase in testicular apoptotic cell death, as well as elevated pro-apoptotic protein levels. Irisin treatment was found to significantly prevent obesity-induced apoptotic effects, which was in agreement with a previous study [35].

AMPKα is a key energy metabolism regulator and has been reported to participate in obesity-related male infertility [21]. Recently, it was found that irisin counteracted high glucose and fatty acid-induced cytotoxicity by AMPKα activation [66]. Irisin could ameliorate high glucose-induced cardiomyocyte injury via the AMPKα signalling pathway [67]. In this study, we also found that the expression and activity of AMPKα were upregulated by irisin treatment in obese mice. After AMPKα inhibition or depletion, the protective effects of irisin on HFD-induced spermatogenesis dysfunction were abrogated, suggesting that the protective effects against obesity-related testicular damage were dependent on activation of AMPKα, which was consistent with previous reports [68–70]. In addition, we also found that irisin-impaired ER stress and irisin-activated Nrf2 were blocked after AMPKα ablation, indicating that the effects of irisin on Nrf2 and ER stress were mediated by AMPKα.

Leydig cells are the sole source of testicular androgens. This type of cell promotes spermatogenesis and sperm transformation via testosterone synthesis. After obesity occurs, the expression of testicular steroid synthesis enzymes and testosterone are reduced by several pathological stimuli. In addition, free saturated fatty acids could also induce Leydig cell apoptosis via the accumulation of oxidation products and the release of ceramide, which further aggravates the reduction in testosterone production induced by obesity [71]. Here, we also found that irisin improved testosterone secretion and increased testicular steroid synthesis enzymes in obese mice. We also found that irisin protected against PA-induced oxidative damage and ER stress in an AMPKα-dependent manner.

However, there are limitations in our study. The irisin receptor was not investigated and still needs further exploration. The precise mechanism by which irisin activates AMPKα was also not documented in this study. Despite the limitations stated above, our study suggested that irisin could improve HFD-induced spermatogenesis deficiency by inhibiting oxidative stress and ER stress. Using AMPKα knockout mice, we also found that the protective effects of irisin were mediated by AMPKα activation. Our study demonstrated that irisin could be a new therapeutic for obesity-related male infertility.

## Conclusions

Considering that the incidence of obesity worldwide continues to increase, finding therapeutic targets that can treat obesity-related male infertility is of crucial importance. We found, for the first time, that irisin administration could attenuate obesity-related spermatogenesis dysfunction in mice by activating the AMPKα signalling pathway. Irisin may be a useful therapeutic antioxidant for protecting against obesity-related male infertility.

## Data Availability

The data that support the findings of this study are available from the corresponding author upon reasonable request.
